# TGF-β Induction of miR-143/145 Is Associated to Exercise Response by Influencing Differentiation and Insulin Signaling Molecules in Human Skeletal Muscle

**DOI:** 10.3390/cells10123443

**Published:** 2021-12-07

**Authors:** Simon I. Dreher, Selina Höckele, Peter Huypens, Martin Irmler, Christoph Hoffmann, Tim Jeske, Maximilian Hastreiter, Anja Moller, Andreas L. Birkenfeld, Hans-Ulrich Häring, Andreas Peter, Johannes Beckers, Martin Hrabě de Angelis, Cora Weigert

**Affiliations:** 1 Department for Diagnostic Laboratory Medicine, Institute for Clinical Chemistry and Pathobiochemistry, University Hospital Tübingen, 72076 Tübingen, Germany; christoph.hoffmann@student.uni-tuebingen.de (C.H.); Andreas.Peter@med.uni-tuebingen.de (A.P.); 2 Institute of Experimental Genetics, Helmholtz Zentrum München, Ingolstädter Landstraße 1, 85764 Neuherberg, Germany; selina.hoeckele@gmail.com (S.H.); peter.huypens@gmail.com (P.H.); martin.irmler@helmholtz-muenchen.de (M.I.); beckers@helmholtz-muenchen.de (J.B.); hrabe@helmholtz-muenchen.de (M.H.d.A.); 3 German Center for Diabetes Research (DZD), 85784 Neuherberg, Germany; Anja.Moller@med.uni-tuebingen.de (A.M.); Andreas.Birkenfeld@med.uni-tuebingen.de (A.L.B.); hu.haering@gmail.com (H.-U.H.); 4 Institute of Bioinformatics and Systems Biology, Helmholtz Zentrum München, Ingolstädter Landstraße 1, 85764 Neuherberg, Germany; tim.jeske@med.uni-muenchen.de; 5 Department of Pediatrics, Dr. von Hauner Children’s Hospital, Ludwig Maximilians Universität, 80337 München, Germany; 6 Institute of Computational Biology, Helmholtz Zentrum München, Ingolstädter Landstraße 1, 85764 Neuherberg, Germany; maximilian.hastreiter@roche.com; 7 Lehrstuhl für Genomorientierte Bioinformatik, Technische Universität München, 85354 Freising, Germany; 8 Institute for Diabetes Research and Metabolic Diseases of the Helmholtz Zentrum München, University of Tübingen, 72076 Tübingen, Germany; 9 Department of Internal Medicine IV, University Hospital Tübingen, 72076 Tübingen, Germany; 10 Chair of Experimental Genetics, Technische Universität München, 85354 Freising, Germany

**Keywords:** exercise, training response, insulin sensitivity, TGF-β1, miR-143, miR-145, IRS1, DEUS

## Abstract

Physical training improves insulin sensitivity and can prevent type 2 diabetes (T2D). However, approximately 20% of individuals lack a beneficial outcome in glycemic control. TGF-β, identified as a possible upstream regulator involved in this low response, is also a potent regulator of microRNAs (miRNAs). The aim of this study was to elucidate the potential impact of TGF-β-driven miRNAs on individual exercise response. Non-targeted long and sncRNA sequencing analyses of TGF-β1-treated human skeletal muscle cells corroborated the effects of TGF-β1 on muscle cell differentiation, the induction of extracellular matrix components, and identified several TGF-β1-regulated miRNAs. qPCR validated a potent upregulation of miR-143-3p/145-5p and miR-181a2-5p by TGF-β1 in both human myoblasts and differentiated myotubes. Healthy subjects who were overweight or obese participated in a supervised 8-week endurance training intervention (*n* = 40) and were categorized as responder or low responder in glycemic control based on fold change ISIMats (≥+1.1 or <+1.1, respectively). In skeletal muscle biopsies of low responders, TGF-β signaling and miR-143/145 cluster levels were induced by training at much higher rates than among responders. Target-mining revealed HDACs, MYHs, and insulin signaling components INSR and IRS1 as potential miR-143/145 cluster targets. All these targets were down-regulated in TGF-β1-treated myotubes. Transfection of miR-143-3p/145-5p mimics in differentiated myotubes validated *MYH1*, *MYH4*, and *IRS1* as miR-143/145 cluster targets. Elevated TGF-β signaling and miR-143/145 cluster induction in skeletal muscle of low responders might obstruct improvements in insulin sensitivity by training in two ways: by a negative impact of miR-143-3p on muscle cell fusion and myofiber functionality and by directly impairing insulin signaling via a reduction in INSR by TGF-β and finetuned *IRS1* suppression by miR-143-3p.

## 1. Introduction

Lifestyle interventions have shown to be very effective in the prevention of type 2 diabetes (T2D) [1], even more effective than metformin treatment [2]. Exercise intervention programs are affordable, technically easy to implement, and have numerous other positive health effects [3]. Physical exercise increases insulin sensitivity [4] and exercise intervention can delay or prevent the onset of T2D and reduce medication intake [5,6,7]. However, there are always individuals who poorly respond to the intervention and who fail to improve in insulin sensitivity or glycemic control [8,9,10]. While the duration, intensity, and mode of exercise significantly influences the individual outcome [11], individual differences are likely responsible for a reduced response in insulin sensitivity after training intervention. The comparison of skeletal muscle transcriptome data of participants in a supervised 8-week endurance exercise intervention study led to TGF-β as a potential regulator of an impaired response in insulin sensitivity [12]. TGF-β has a central role in skeletal muscle development, tissue inflammation, and regenerative processes [13]. Prolonged endurance exercise and resistance training up-regulates TGF-β expression in human skeletal muscle [14,15], which is suggested to play a role in muscle repair mechanisms [16]. Chronic elevation or activation of TGF-β impairs differentiation of myoblasts [17], triggers tissue fibrosis during chronic inflammation in muscular dystrophies [16], and reduces insulin signaling and expression of metabolic regulators (such as PGC1α) [12,18].

The effects of TGF-β are mediated after cell membrane receptor binding by the activation of intracellular signaling cascades which also regulates epigenetic mechanisms. Several studies showed effects of TGF-β on miRNAs [19,20,21,22] or the miRNA machinery [23]. With the growing understanding of miRNAs, it became clear that expression patterns of miRNAs are tissue-specific. Skeletal muscle-specific miRNAs have been implicated in the development of skeletal muscle and the regulation of myogenic differentiation [24,25]. During this process, skeletal muscle-derived myoblasts fuse to form myotubes, which is a tightly controlled process easily disturbed by dysregulation of potent differentiation regulators, such as TGF-β [12,26]. Very recently, induction of miRNAs in skeletal muscle has been implicated in improved glucose metabolism after endurance exercise [27]. On the other hand, elevated levels of certain miRNAs have been involved in the reduction in insulin sensitivity in liver and adipose tissue, including TGF-β-regulated miR-143 [28,29,30,31,32,33,34].

We, thus, hypothesized that one arm of the TGF-β effect on exercise response and metabolic regulators in skeletal muscle acts through the regulation of miRNA expression patterns. Therefore, we analyzed the TGF-β1-regulated transcriptome, including miRNAs and long RNAs in fully fused primary human myotubes previously treated with TGF-β1 at the myoblasts state. To consider the impact of TGF-β1 on differentiation of skeletal muscle cells, we analyzed the regulation of the identified miRNAs in myoblasts and fully differentiated myotubes. We further investigated the identified TGF-β1-regulated miRNAs in vivo in human skeletal muscle biopsies and blood samples obtained from subjects classified as responders and low responders in insulin sensitivity after a supervised 8-week endurance exercise intervention. After target gene mining, combining in vivo and in vitro with in silico approaches, we investigated the role of TGF-β1 and exercise-response associated miRNAs on cell differentiation, insulin signaling, and metabolic regulators. Thereby, we aim to elucidate the potential impact of TGF-β1-driven miRNAs on the individual exercise response.

## 2. Materials and Methods

### 2.1. Primary Cell Culture

Human satellite cells were obtained by percutaneous needle biopsies performed on *vastus lateralis* muscle. Cells were released by collagenase digestion and seeded on 15-cm dishes coated with GelTrex (thin layer protocol, 1:300, Life Technologies, Waltham, MA, USA). After two rounds of proliferation in cloning medium (39% α-MEM, 39% Ham’s F-12, 20% FBS, 1% chicken extract, 100 U/mL penicillin, 100 μg/mL streptomycin and 0.5 μg/mL amphotericin B), CD56-positive myoblasts were enriched using MACS microbeads and LS columns (Milteny Biotech, Bergisch-Gladbach, Germany), according to the manufacturer’s protocol, with a 30-min incubation. They were then stored in the gaseous phase of liquid nitrogen. For experiments, cells were seeded at 1000 cells/cm^2^ in cloning medium on 6-well plates. Myotube differentiation was initiated by either 2% FBS or FBS-free fusion medium (α-MEM, 100 U/mL of penicillin, 50 μM of palmitate, 50 μM of oleate, 100 μM of carnitine, 100 μg/mL of streptomycin, and 0.5 μg/mL of amphotericin B) and continued for 5 days. Stimulations with TGF-β1 were performed for 48 h before initiation or during myotube differentiation for 96 h or 48 h, as indicated. Human TGF-β1 (R&D Systems, Minneapolis, MN, USA) was dissolved in 4 mM of HCl 0.2% BSA while the ALK4/5/7 inhibitor SB431542 (Merck, Darmstadt, Germany) was solved in DMSO. Furthermore, 4 mM of HCl 0.2% BSA served as control. Differentiated myotubes were transfected with miRNA mimics (hsa-miR-143-3p miRCURY LNA miRNA Mimic, hsa-miR-145-5p miRCURY LNA miRNA Mimic; Qiagen, Hilden, Germany) for 24 h using Lipofectamine (RNAiMax, Thermo Fischer, Waltham, MA, USA), according to the manufacturer’s instructions, and were analyzed after 48 or 96 h. All miRNA mimics were transfected at a final concentration of 12.5 nM. Transfection reagents without mimic, hsa-miR-122-5p miRCURY LNA mi RNA mimic (Qiagen)), and negative control miRCURY LNA miRNA mimic, served as controls.

### 2.2. qPCR

Total RNA was extracted by employing the NucleoSpin miRNA kit (Macherey-Nagel, Düren, Germany). For mRNA/miRNA detection, reverse transcription was carried out with the Transcriptor First Strand Synthesis Kit (Roche, Basel, Switzerland). Expression was measured using QuantiFast SYBR Green PCR Mix and QuantiTect Primer Assays (Qiagen, Table S2) in a LightCycler 480 (Roche). mRNA standards for PCR were generated by purifying PCR-Product (MinElute PCR Purification Kit, Qiagen) and 10-fold serial dilution. Alternatively, for microRNA detection, the TaqMan MicroRNA RT Kit Cat#: 4,366,596 and TaqMan MicroRNA Assays Cat#: 4,427,975 (Thermo Fisher) were used according to manufacturer’s protocols (Table S2).

### 2.3. Immunoblot

Cells were lysed with 200 μL of RIPA buffer (25 mM of Tris, 150 mM of NaCl, 3.5 mM of SDS, 12.06 mM of NaDOC, 14.44 mM of TritonX100) containing protease- (1× cOmplete EDTA-free protease inhibitor cocktail, Merck) and phosphatase inhibitors (1 mM of NaF, 0.5 mM of Na-phyrophosphate, 1 mM of ß-glycerophosphate, 1 mM of Na-orthovanadate) at pH 7.6 and protein concentration was quantified with BCA-Assay (Pierce Biotechnology, Waltham, MA, USA). Proteins were separated by sodium dodecylsulfate polyacrylamide (7.5–15%) gradient gel electrophoresis and transferred onto a nitrocellulose membrane (GE Healthcare, Amersham, Great Britain) by semi-dry electroblotting. Blocking was performed in an NET buffer (150 mM of NaCl, 50 mM of Tris/HCl, pH 7.4, 5 mM of EDTA, 0.05% Triton X-100, and 0.25% gelatine). The primary antibodies (Table S2) were added in NET overnight at 4 °C or for 2 h at room temperature. Fluorescence-labeled secondary antibodies (LI-COR Biosciences, Bad Homburg, Germany, Table S2) were added (1 h, room temperature). Detection was performed on an Odyssey scanner (LI-COR Biosciences).

### 2.4. Sequencing

After total RNA isolation, for long RNAs, the Ovation RNA-Seq System V2 for cDNA synthesis, amplification, and purification was used; and, for sncRNAs, the NEBNext Small RNA Library Prep Set for Illumina was used. cDNA was fragmented to a size of 200 bp using the Covaris system. Libraries were generated using the NEBNext Ultra DNA Library Prep Kit for Illumina. Libraries were tested during processing with a Nanodrop spectrophotometer and bioanalyzer. The same controls of quality and quantity were used for final products. Sequencing of myotube RNA was performed on a HiSeq 2500 (Illumina, San Diego, CA, USA) using HiSeq PE Rapid Cluster Kit v2, HiSeq Rapid SBS Kit v2 (200) and HiSeq Rapid SBS Kit v2 (50 cycles), all from Illumina for the 2 × 125 bp paired-end sequencing analysis of long RNAs. For small RNAs, the HiSeq Rapid SR Cluster Kit v2 and HiSeq Rapid SBS Kit v2 (50 cycles) for single-read 1× 50 bp sequencing were used. FastQ files were generated with CASAVA BCL2FASTQ Conversion Software. Small RNAseq data were preprocessed with Trim Galore (v0.0.4, http://www.bioinformatics.babraham.ac.uk/projects/trim_galore/) and aligned with read mapper STAR v2.5.2a. Long RNAseq data were preprocessed with Trim Galore (v0.6.6, http://www.bioinformatics.babraham.ac.uk/projects/trim_galore/) and aligned with read mapper STAR v2.7.6a. Sequencing data were submitted to the GEO database at NCBI (GSE188236). Small RNA sequencing data were analyzed by two approaches, as published in [35]. In brief, in the classical approach, sequences were annotated to the genome before comparing testing conditions. In the DEUS (differential expression of unique sequences) analysis, raw sequences of testing conditions were compared prior to annotation to the genome.

### 2.5. Exercise Intervention Studies

The analyses performed in this manuscript were based on two recently published exercise intervention studies. Participants and design of the two studies were described recently [12,36] (NCT03151590 at Clinicaltrials.gov). Healthy but overweight individuals with a high risk for T2D: <120 min of physical activity per week and at least one of the following risk factors (BMI > 27 kg/m^2^, family history of T2D, former gestational diabetes) were recruited (Table 1). In both studies, the design of the exercise intervention and criteria for recruitment were identical. In brief, the participants performed an 8-week supervised endurance-exercise training, as detailed below. Individual training intensity was set at 80% of VO_2_peak, assessed by metabolic gas analysis (MetaLyzer 3B and MetaMax 3B, Cortex Biophysics GmbH, Leipzig, Germany), before the intervention and controlled by heart rate corresponding to 80% VO_2_peak. Each session consisted of 30 min on the treadmill and 30 min on a bicycle. Insulin sensitivity was assessed after an overnight-fast by 75 g-OGTT pre-intervention and 5 days after the last exercise bout. The insulin sensitivity index was estimated by the method of Matsuda and DeFronzo (ISI_Mats_) [37]. Whole blood samples for isolation of RNA were obtained in the fasted state. Muscle biopsies were obtained from the lateral part of *vastus lateralis* muscle before and after the training period. Total RNA was isolated from snap-frozen muscle biopsies after homogenization (TissueLyser II, Qiagen) using the miRNeasy Kit (Qiagen), including DNAse digestion. In study 1, total RNA was amplified using the Ovation Pico WTA System V2 in combination with the Encore Biotin Module (NuGEN Technologies Inc., San Carlos, CA, USA). Amplified cDNA was hybridized on Affymetrix Human Transcriptome Array 2.0 (Affymetrix, Santa Clara, CA, USA), as described in [12]. In study 2, total RNA was amplified using the WT PLUS Reagent Kit (Thermo Fisher). Amplified cDNA was hybridized on Human Clariom S arrays (Thermo Fisher). Staining and scanning (GeneChip Scanner 3000 7G) was performed according to manufacturer’s instructions. Transcriptome Analysis Console (TAC; version 4.0.0.25; Thermo Fisher)) was used for quality control and to obtain annotated normalized SST-RMA gene-level data for both arrays. Statistical analyses were performed with R3.6.3/Rstudio [38]. Array data were submitted to the GEO database at NCBI (GSE72462 and GSE161749). In total, array data and samples of 40 individuals (17 from study 1 and 23 from study 2) were included in the present study. All participants gave written informed consent and the study protocols were approved by the ethics committee of the University of Tübingen and in accordance with the declaration of Helsinki.

### 2.6. Statistical Analyses

Data obtained from both exercise intervention studies were combined and linear regression models analyzed with R3.6.3/RStudio. Normality was tested by the Shapiro–Wilk-test from the R package ‘stats’ (v3.6.3), and non-normal data were log-transformed. Genewise testing for differential gene expression between time points and response groups were calculated by employing the (paired) limma *t*-test with the R package ‘limma’ (v3.46.0). To reduce background, gene sets were filtered using detection above background *p*-values < 0.05 in more than 50% of the samples in at least one of the treatment groups per comparison. All data were given as mean ± SD. Differences between groups were assessed using one-way ANOVA with Fisher’s LSD post hoc test or Bonferroni correction for multiple comparisons where appropriate. Comparisons between several components per group were made using the paired linear mixed model. Graphs were made using the R packages ‘ggplot2’ (v3.3.2) and ‘ggrepel’ (v0.9.1), and heatmaps were generated using GraphPad Prism (v9.1.2) and assembled using InkScape (v1.0). Functional over-representation analysis was carried out online using https://biit.cs.ut.ee/gprofiler/ (accessed on 28 October 2021) with *p* < 0.05 as threshold and Bonferroni correction for multiple testing. Homo sapiens was chosen as an organism and analysis was performed for gene ontology biological process (GO:BP).

## 3. Results

### 3.1. TGF-β1 Modulates Global miRNA Expression Profile in Differentiating Human Skeletal Muscle Cells

**Figure 1:**
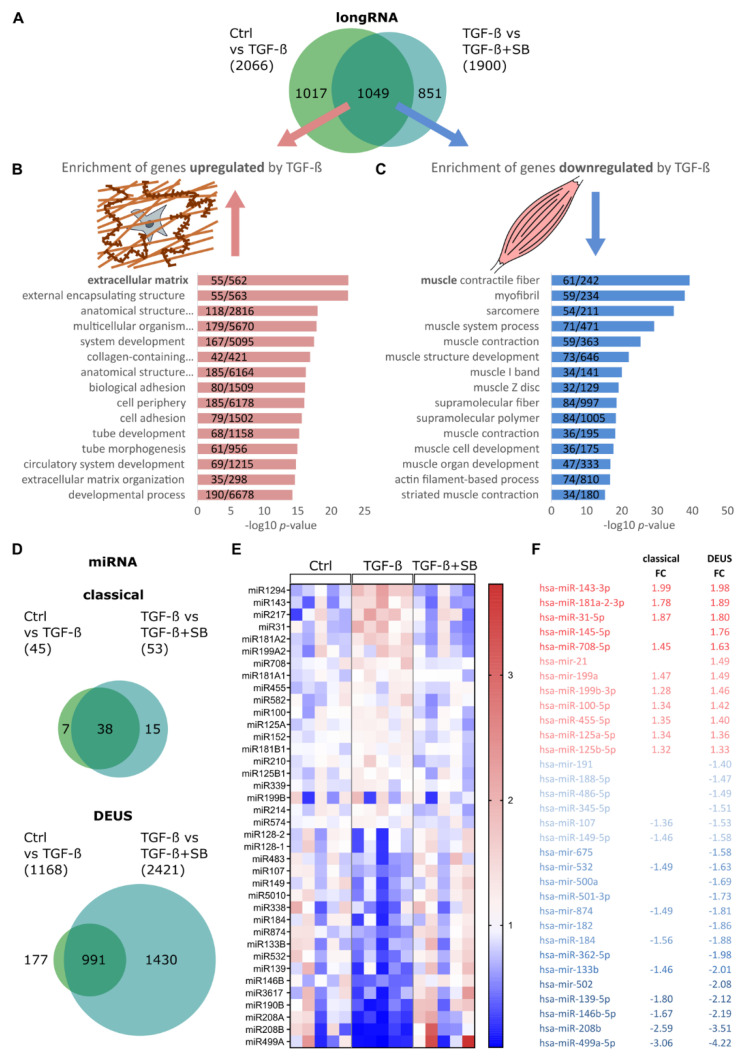
TGF-β1 modulates global RNA expression profile in differentiating human skeletal muscle cells. Primary human myoblasts (*n* = 5) were treated with 1 ng/mL of TGF-β1, TGF-β1 plus inhibitor (10 µM SB431542), or vehicle control for 48 h. Subsequently, myoblasts were differentiated towards myotubes for 5 days in the presence of 2% FBS. RNA samples underwent sequencing. (**A**) Differentially regulated long RNA transcripts with a FC > |1.3| (*p* < 0.05) are shown. The overlap of 1049 transcripts differentially regulated by TGF-β1 and rescued by combination with the inhibitor was stratified in up- (**B**) or downregulated (**C**) by TGF-β1 and resulting lists submitted to GO enrichment analysis for biological processes. The top 15 significantly enriched processes (*p* < 0.05 Bonferroni corrected) are shown and the number of represented genes/all genes in the term are indicated. (**D**) Differentially regulated sncRNA transcripts with a FC > |1.3| (*p* < 0.05) are shown. Sequencing data were analyzed by two distinct approaches. The classical pipeline annotates sequences to the gene names of sncRNAs, followed by evaluation of differential regulation. The DEUS analysis evaluates unique sequences for differential regulation before annotation. (**E**) The overlap of the 38 differentially regulated miRNA transcripts based on the classical approach is represented by a heatmap. Colors represent average-normalized values. (**F**) Overlap of average linear FC for each annotated miRNA by the DEUS analysis with differentially regulated miRNAs detected by the classical approach.

To characterize the general impact of TGF-β1 on gene and miRNA expression in an unbiased manner, we sequenced total long (RNAseq) and small (miRseq) RNA after 5 days of myo-differentiation. Primary human skeletal muscle cells were pre-confluently stimulated for 48 h with 1 ng/mL of TGF-β1, or additionally with 10 µM of SB431542, an ALK4/5/7 inhibitor that prevents canonical TGF-β signaling via phosphorylation of SMAD3 [39], and subsequently differentiated. In total long RNAseq, we found 2066 transcripts differentially regulated (*p* < 0.05, FC > |1.3|) in TGF-β1-treated samples compared to vehicle-treated control. Compared to TGF-β1 only, 1900 transcripts were differentially regulated when treated with the combination of TGF-β1 and the inhibitor. In the overlap, all 1049 regulated transcripts showed an opposite regulation in samples treated with TGF-β1 and the inhibitor combination (Figure 1A). The list of overlapping, TGF-β1-specific regulated sequences was separated in up- or downregulated transcripts (424 and 625 transcripts, respectively). Genes specifically upregulated by TGF-β1 were enriched in terms associated with extracellular matrix (ECM) and developmental processes while downregulated genes were specifically enriched in terms associated with muscle function and development (Figure 1B,C). Thus, as expected, treatment of myoblasts with TGF-β1 exerts long-lasting effects on ECM production while it negatively impacts muscle cell differentiation.

Small RNA sequencing (miRseq) data underwent two different analysis approaches. In the classical approach, the comparison of vehicle control and TGF-β1 treated cells led to 45 differentially regulated annotated miRNAs (*p* < 0.05, FC > |1.3|). Of these, 38 miRNAs showed an opposite regulation in TGF-β1 plus inhibitor-treated cells (Figure 1D,E). In contrast, by DEUS analysis, the comparison of vehicle-control and TGF-β1 treated samples yielded 1168 differentially regulated unique sequences (*p* < 0.05, FC > |1.3|) and the overlap with opposite regulated unique sequences in the TGF-β1 and inhibitor-treated condition contained 991 unique sequences (Figure 1D). These unique sequences often share identical regions with other unique sequences [35], which explains the considerably higher numbers.. About 60% of the unique sequences from DEUS analysis cover annotated sncRNA. To identify the most robust TGF-β-regulated miRNAs, we only focused on miRNAs for which the differential expression after TGF-β1 treatment was reversible with TGF-β1 plus inhibitor treatment. Most miRNAs identified by the classical approach could be validated by DEUS analyses. With DEUS, a higher specificity was achieved than with the classical pipeline, distinguishing between -3p and -5p versions of mature miRNAs in many cases (Figure 1F). The top miRNAs most upregulated by TGF-β1 treatment were miR-143-3p, miR-181a2-3p, miR-31-5p, and miR-145-5p. The top most downregulated miRNAs were miR-499a-5p, miR-208b, miR146b-5p, and miR-139-5p. This global sequencing approach showed that TGF-β has a substantial effect on the miRNA transcription profile of differentiating skeletal muscle cells and yielded candidate miRNAs for further analysis.

### 3.2. TGF-β1 Regulates miR-143/145 Cluster and miR-181a2 Differentiation-Independantly

Since we investigated the TGF-β1 effects on global miRNA expression in differentiating myotubes and found a profound impact of TGF-β1 on the differentiation of these cells, we next studied the impact of differentiation on our candidate miRNAs. Human myoblasts were cultured until confluency and subsequently differentiated into myotubes for 5 days.

**Figure 2:**
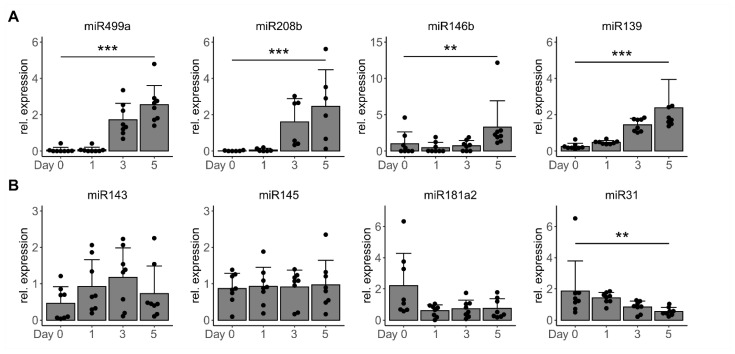
Expression of TGF-β-regulated miRNAs during skeletal muscle cell differentiation. Primary human myoblasts (*n* = 8) were differentiated towards myotubes for 5 days without FBS. Day 0 equals the start of differentiation by serum withdrawal. Expression of mature miRNAs was analyzed by qPCR on day 1, 3, and 5 of the differentiation process. (**A**) miRNAs identified as downregulated by TGF-β1 treatment of myoblasts. (**B**) miRNAs identified as upregulated by TGF-β1 treatment of myoblasts. All qPCR data were normalized to individual donors and reference RNU6. Individual datapoints are displayed, bars represent mean ± SD. All data were analyzed using the Kruskal test for trend. ** *p* < 0.01, *** *p* < 0.001.

For this and all the following experiments, the chosen differentiation protocol was adjusted to 0% FBS which yielded an improved myotube differentiation rate [26], also shown by elevated muscle-specific miR-499a-5p, miR-208b and differentiation marker *MYH4* comparing differentiation in the presence of 2 and 0% FBS (Figure S1A,B,K). The effects of TGF-β1 when added to pre-confluently myoblasts on differentiation and our candidate miRNAs were also validated in the myotubes differentiated in 0 % FBS except for miR-146b-5p. (Figure S1). *MYH1* was not significantly regulated in myotubes when TGF-β1 was added to the myoblasts (Figure S1I). Next, miRNA expression was analyzed on days 0, 1, 3, and 5 of the differentiation process. As expected for muscle-specific miRNAs, such as miR-499a-5p and miR-208b, all top-candidate miRNAs identified as downregulated by TGF-β1 were significantly upregulated during myotube differentiation (Figure 2A,B). Levels of miR-31-5p were downregulated. Thus, regulation of these miRNAs by TGF-β could not be distinguished from TGF-β’s effect on differentiation itself. On the contrary, miR-143-3p, miR-145-5p, and miR-181a2-3p were not significantly regulated during myotube differentiation. Specific upregulation of these candidate miRNAs by TGF-β1 was further validated in undifferentiated myoblasts (Figure S2A–C). All candidates were strongly and significantly induced already 48 h after TGF-β1 stimulation which was prevented by the addition of the inhibitor. These data together indicate a strong and specific induction of miR-143-3p, miR-145-5p, and miR-181a2-5p by elevated TGF-β levels in human skeletal muscle cells, identifying them as the most promising TGF-β-driven miRNA candidates in human skeletal muscle cells.

### 3.3. Low Responders Show Elevated TGF-β Signaling and miR-143/145 Cluster Induction by Training

**Figure 3:**
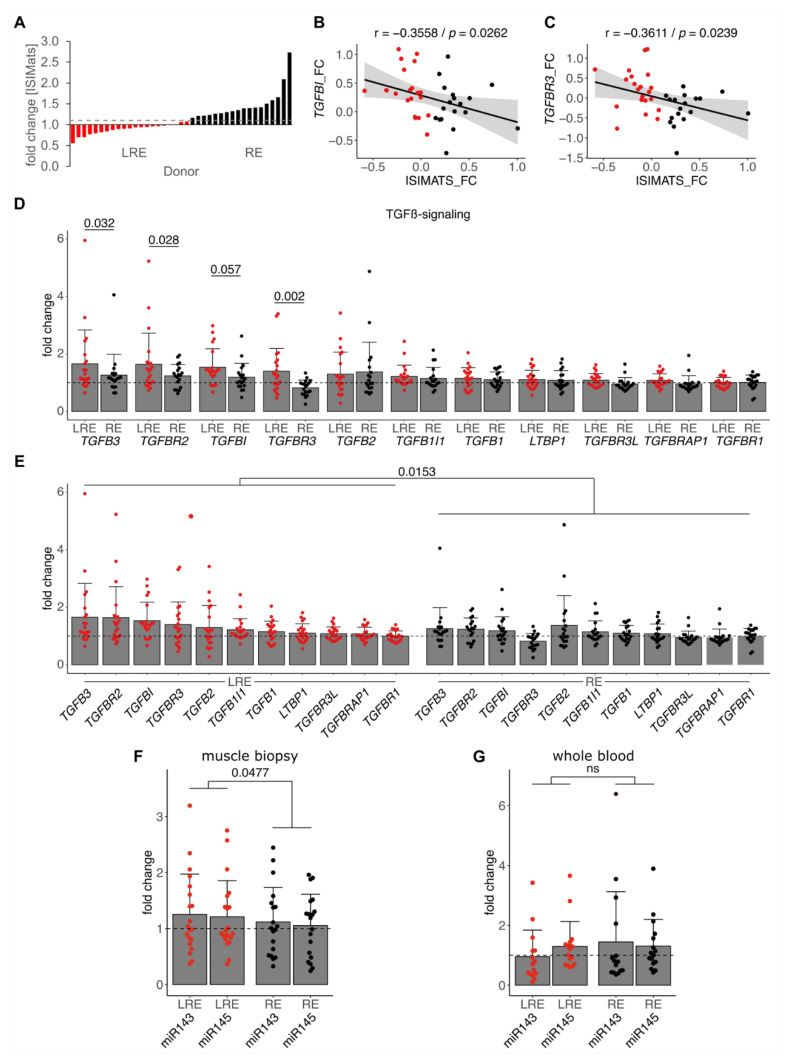
Low responders show elevated TGF-β signaling and miR-143/145 cluster induction by training. (**A**) Middle-aged sedentary individuals (*n* = 40) were grouped into low responders (LRE, red) and responders (RE, black) based on their improvement (fold change > 1.1) in insulin sensitivity (ISIMats) after 8 weeks of training intervention. RNA isolated from muscle biopsies before and after training was used for transcriptome analyses. (**B**,**C**) Correlation analyses of log-transformed fold changes (after intervention vs. before intervention) in ISIMats and fold changes of TGF-β target gene *TGFBI* and *TGFBR3* transcripts in muscle biopsies was performed. (**D**,**E**) Fold change expression (after intervention vs. before intervention) of genes annotated in the term TGF-β signaling (GO:0007179) and detected by transcriptome analyses was compared between LREs and REs. Expression of mature miRNA cluster miR-143/145 was analyzed in skeletal muscle biopsies (**F**) and whole blood (**G**) by qPCR, normalized to individual donors and reference RNU6. Individual datapoints are displayed, bars represent mean ± SD. Data were analyzed using Pearson correlation (**B**,**C**), one-way ANOVA with Fisher’s LSD post-hoc test (**D**) or a paired linear mixed model (**E**–**G**). *p*-values are indicated.

Next, we analyzed transcripts related to TGF-β signaling and TGF-β-regulated miRNAs in skeletal muscle of responders (RE) and low responders (LRE) based on insulin sensitivity after an 8-week exercise intervention [12,36]. The cohort consisting of *n* = 40 individuals was stratified according to their training response, defined as an improvement in insulin sensitivity (ISIMats) with a cutoff at 1.1 (Figure 3A). As expected, and similar to the previous report [12], a negative training response marked by a low fold change in ISIMats correlated with a high fold change in TGF-β signaling marker *TGFBI* and *TGFBR3* expression in muscle biopsies of the combined cohort (Figure 3B,C). We next investigated regulation of all detected transcripts annotated in the term TGF-β signaling (GO:0007179) between LRE and RE. Many TGF-β signaling components were significantly stronger induced by training in LREs compared to REs (Figure 3D). Paired analysis over all components revealed a significantly stronger induction of TGF-β signaling by training in LREs (Figure 3E). These analyses again confirmed the detrimental connection of elevated TGF-β signaling and training response in ISIMats. As we identified, candidate miRNAs regulated by TGF-β in skeletal muscle cells confirmed the connection between an impaired training response and elevated TGF-β signaling in human subjects. Next, we analyzed the miRNA expression in the skeletal muscle biopsies of these individuals. While potential candidate miR-181a2-5p was not differentially induced between the response groups (Figure S3A), the miR-143/145 cluster was significantly induced in skeletal muscle of LREs by training compared to REs (Figure 3F). Similar to systemic levels of TGF-β [12], the changes in systemic miR-143/145 cluster levels were not different between the response groups in corresponding blood samples (Figure 3G). Thus, the stronger induction of TGF-β signaling as well as of the miR-143/145 cluster by training was observed locally in skeletal muscle tissue of low responders, but not on a systemic level.

### 3.4. Target Mining for Potential Skeletal Muscle Specific miR-143/145 Cluster Targets

**Figure 4:**
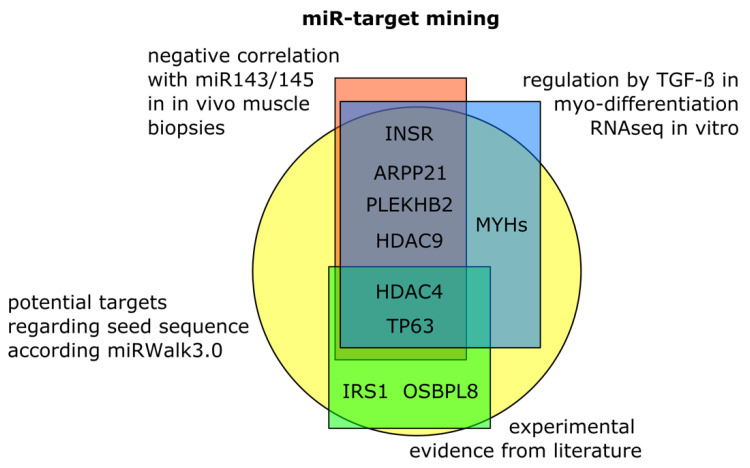
Target mining for potential skeletal muscle specific miR-143/145 targets. The following criteria were applied: transcripts had to contain a potential seed sequence for either miR-143-3p or miR-145-5p based on the MirWalk3.0 tool [40] (yellow circle); target candidates had to be downregulated by TGF-β1 treatment and rescued by combining TGF-β1 and inhibitor treatment in differentiating myotubes in vitro as detected by long RNASeq (blue rectangle); and potential targets had to show a negative correlation in a global correlation analysis between the changes in miR-143-3p and miR-145-5p, as well as the transcript detected by transcriptome analyses in the in vivo muscle biopsies (red rectangle). Additional targets were considered where previous experimental evidence as a target of miR-143/145 in related systems exists in the literature [31,32,33] (green rectangle).

To elucidate how the altered miRNA expression is connected to the differences in the response to training, all generated datasets were mined for potential targets of miR-143-3p or miR-145-5p utilizing a weighted integrative approach. The following criteria were applied: transcripts had to contain a potential seed sequence for either miR-143-3p or miR-145-5p based on the MirWalk3.0 tool [40] (Figure 4); target candidates had to be downregulated by TGF-β1 treatment and rescued by combining TGF-β1 and inhibitor treatment in differentiating myotubes in vitro as detected by long RNASeq; and, finally, a potential target had to show a negative correlation in a global correlation analysis between the changes in miR-143-3p or miR-145-5p, as well as the transcript detected by transcriptome analyses in the in vivo muscle biopsies (Figure S4). INSR, ARPP21, PLEKHB2, HDAC4/9, and TP63 met all three criteria (Figure 4). IRS1 and OSBPL8 were also considered because previous experimental evidence as a target of the miR-143/145 cluster exists in the literature [31,32,33]. In addition, myosin heavy chains (MYH) were considered because they were regulated in myotubes by TGF-β1 and contain a potential seed sequence for miR-143/145. The data suggest a direct effect of miR-143/145 cluster on transcriptional status, muscular function, and insulin signaling in human skeletal muscle cells.

### 3.5. TGF-β Negatively Impacts Functional Components and Insulin Signaling in Skeletal Muscle Cells

The next step was to evaluate which of the identified potential targets in Figure 4 were regulated by TGF-β-driven miR-143/145 cluster in fully differentiated myotubes.

**Figure 5:**
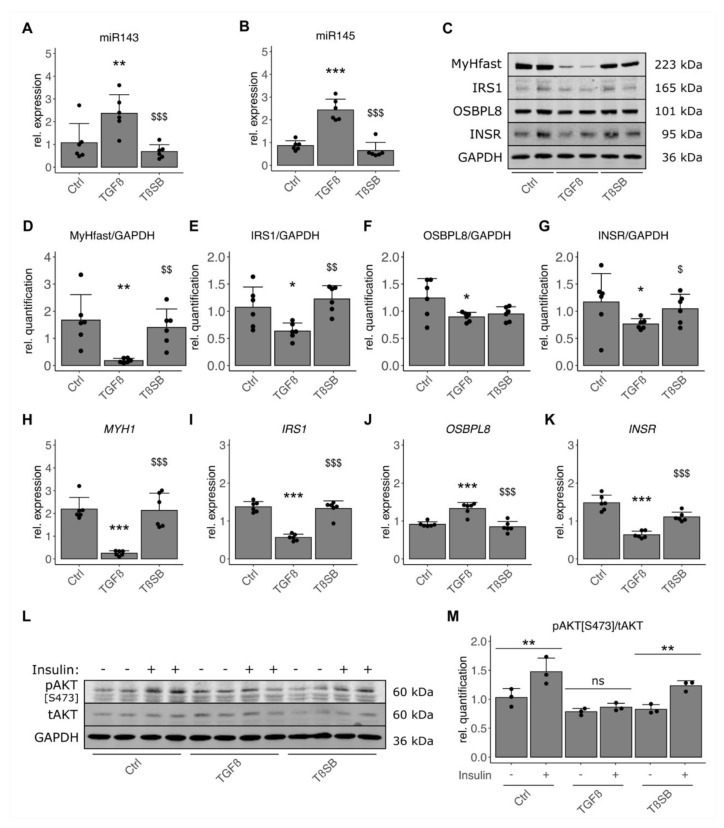
TGF-β1 negatively impacts potential miR-143/145 cluster targets and insulin signaling in human skeletal muscle cells. Primary human myoblasts (*n* = 6) were differentiated towards myotubes for 5 days in the absence of FBS and subsequently treated with 1 ng/mL of TGF-β1 (TGFβ), TGF-β1 plus inhibitor (10 µM of SB431542) (TβSB), or vehicle control (Ctrl) for the last 48 h. Expression of mature miRNA (**A**) miR-143-3p, (**B**) miR-145-5p was detected by qPCR. (**C**) Protein levels of MYH1/2 (MyHfast), IRS1, OSBPL8, and INSR were detected by Western Blot and quantified (**D**–**G**) by normalizing to internal loading control GAPDH. mRNA levels of (**H**) *MYH1*, (**I**) *IRS1*, (**J**) *OSBPL8*, and (**K**) *INSR* were detected by qPCR. All qPCR data were normalized to individual donors and reference RNU6 (miRNAs) or mean of *RPS28* and *TBP* (mRNA). (**L**) Insulin-induced phosphorylation of AKT (pAKT[S473]), total AKT (tAKT), and GAPDH were detected by Western Blot after an additional 10-min stimulation with 10 nM of insulin (*n* = 3). (**M**) The ratio of pAKT per tAKT was calculated after quantification. Individual datapoints are displayed, bars represent mean ± SD. All data were analyzed using one-way ANOVA with Fisher’s LSD post-hoc test. (**A**–**K**) * *p* < 0.05, ** *p* < 0.01, *** *p* < 0.001 vs. Ctrl, $ *p* < 0.05, $$ *p* < 0.01, $$$ *p* < 0.001 vs. TGFβ, (**M**) * *p* < 0.05, ** *p* < 0.01, *** *p* < 0.001 vs. no insulin (−).

Human primary myoblasts were cultivated and differentiated for 5 days into multinucleated myotubes, and treated with 1 ng/mL of TGF-β1 or TGF-β1 and 10 µM of SB431542 for the last 48 h. Even in fully differentiated myotubes, TGF-β1 treatment elevated miR-143-3p/145-5p levels (Figure 5A,B). TGF-β1 also reduced most of the identified potential targets in differentiated human skeletal muscle cells (Table 2), in particular myosin heavy chain expression as well as INSR and IRS1 on mRNA and protein level (Figure 5C–K). All of these effects were reversed by treating in combination with the inhibitor.

However, *PLEKHB2* was not affected by the treatment and OSBPL, previously described as miR-143/145 target in other tissues [31,32], was even elevated on mRNA, yet unaffected on protein level in our human skeletal muscle cells (Table 2, Figure 5C,F,J). Additional stimulation with insulin for 10 min revealed that TGF-β1 pretreatment prevented phosphorylation of Ser-473 of AKT completely (Figure 5L,M). Together, this shows that TGF-β elevated miR-143-3p/145-5p levels and suppressed myo-essential proteins, such as MYH1, as well as insulin signaling potentially via a reduction in signaling components INSR and IRS1.

### 3.6. miR-143/145 Cluster Finetunes TGF-β Effects on Differentiation and Insulin Signaling

**Figure 6:**
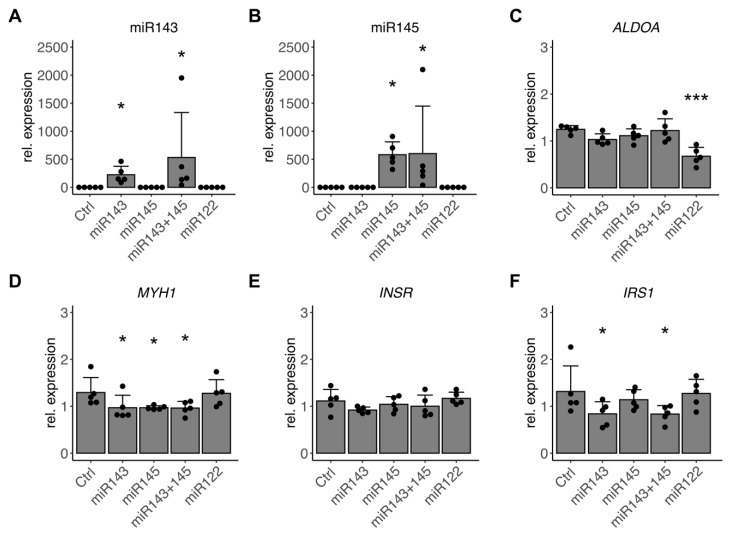
miR-143/145 downregulate components of skeletal muscle differentiation and insulin signaling. Primary human myoblasts (*n* = 5) were differentiated towards myotubes for 5 days in the absence of FBS and subsequently transfected with miR-143-3p, miR-145-5p, a combination of miR-143-3p and miR-145-5p, control miR-122-5p, or vehicle control (Ctrl) for the last 48 h. Abundance of mature miRNA (**A**) miR-143-3p, (**B**) miR-145-5p was detected by qPCR. (**C**–**F**) mRNA levels of (**C**) miR-122-5p target *ALDOA*, (**D**) *MYH1*, (**E**) *INSR*, and (**F**) *IRS1* were detected by qPCR. All qPCR data were normalized to individual donors and reference RNU6 (miRNAs) or mean of *RPS28* and *TBP* (mRNA). Individual datapoints are displayed, bars represent mean ± SD. All data were analyzed using one-way ANOVA with Fisher’s LSD post-hoc test, * *p* < 0.05, *** *p* < 0.001 vs. Ctrl.

Finally, to titrate the specific contribution of miR-143/145 cluster from the observed impact of TGF-β on human skeletal muscle cells, we transfected miR-143-3p, miR-145-3p, and a combination of both directly in differentiating myotubes. After 48 h and 96 h, strongly and significantly elevated levels of miR-143-3p and miR-145-5p were measured in respective groups (Figure 6A,B and Figure S5A,B). A significant reduction in described target *ALDOA* by control miR-122-5p served as proof of concept and marked 48 h as the most valuable timeframe for readouts (Figure 6C and Figure S5C). Elevation of either miR-143-3p or miR-145-5p alone as well as in combination significantly reduced *MYH1* and *MYH4* expression (Figure 6D; Table 3). While *INSR* was not affected by miR-143/145 cluster elevation (Figure 6E, Table 3), increased levels of miR-143-3p significantly reduced *IRS1* expression (Figure 6F, Table 3). After 96 h, the effect on *IRS1* was not significant anymore while the reduction in *MYH1* expression by miR-143-3p prevailed (Figure S5D–F, Table S1). Other potential targets, such as *OSBPL8*, remained unaffected by elevated miR-143-3p or miR-145-5p in human skeletal muscle cells (Table 3 and Table S1). Potential targets, such as *ARPP21* or *TP63*, were regulated after 96 h only but not consistently by a specific miRNA (Table 3 and Table S1).

In summary, this suggests that the observed effect of TGF-β on myosin heavy chains is, in part, mediated by its regulation of the miR-143/145 cluster and that the suppression of insulin signaling is finetuned by miR-143-3p, which transiently suppresses *IRS1*.

## 4. Discussion

To understand the role of TGF-β in the metabolic adaptation to exercise, the mechanisms by which TGF-β influences metabolic regulators and insulin signaling need to be elucidated. Since our previous data demonstrate a sustained regulation of TGF-β target genes in skeletal muscle biopsies after the 8-week training intervention, we focused on TGF-β-driven miRNAs as a long-lasting epigenetic mechanism and studied their function in skeletal muscle differentiation and individual exercise response. We started with an unbiased approach by profiling TGF-β effects and regulated miRNAs considering human skeletal muscle cell differentiation as a relevant contributing factor.

**Figure 7:**
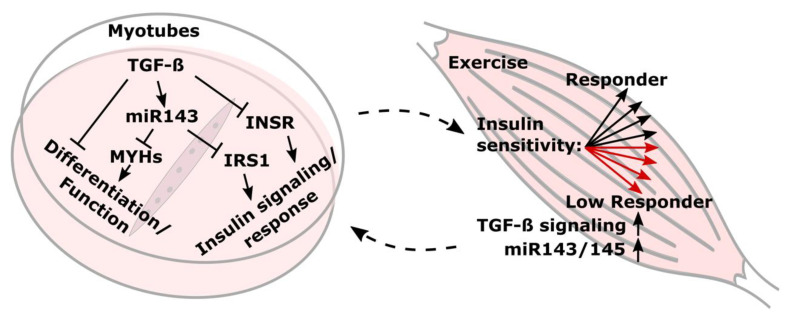
TGF-β-induced miR-143/145 influences differentiation, insulin signaling, and exercise response in human skeletal muscle.

TGF-β1 treatment of myoblasts impaired myotube differentiation and drove miR-143, miR-145, and miR-181 differentiation independently. We confirmed that the elevated TGF-β signaling is negatively associated with a beneficial training response in metabolic control in overweight human individuals. An improvement in insulin sensitivity (ISIMats) after an 8-week exercise intervention allowed for categorization of participants in responders and low responders. In muscle biopsies of low responders, TGF-β signaling and the miR-143/145 cluster were induced by training (Figure 7). A target mining approach yielded a list of potential targets of miR-143/145 in skeletal muscle cells of which *MYH1*, *MYH4*, and *IRS1* could be validated. TGF-β upregulated the miR-143/145 cluster and suppressed myosin heavy chains, INSR, IRS1, and insulin signaling in fully differentiated myotubes. miR-143 directly reduced *MYH1/4* and finetuned *IRS1* suppression in differentiated myotubes contributing to reduced insulin signaling and presumably muscular performance. In this study, we, for the first time, described how TGF-β affected skeletal muscle cells via regulation of the miR-143/145 cluster and suggest that parts of the TGF-β effects are mediated via miR-143 regulating *IRS1* and the novel targets *MYH1* and *MYH4* (Figure 7).

TGF-β signaling is capable of modulating miRNA expression at both the transcriptional and post-transcriptional level via R-SMADs. The most common miRNAs upregulated by TGF-β signaling include miR-21, the miR-181 family, miR-10b, the miR-17/92 cluster, miR-155, miR-192, the miR-23/24/27 cluster, miR-216/217, miR-494, and miR-182. The miRNAs downregulated by TGF-β signaling include the miR-200 family, miR-203, miR-34a, and miR-584, as reviewed by Suzuki in 2018 [28]. While, from this list, we also identified miR-181a2 as a TGF- β regulated miRNA in skeletal muscles cells in vitro, it was not regulated by training in skeletal muscle of either responders or low responders in vivo. Others have shown an increase in miR-143/145 expression after TGF-β treatment in non-small cell lung cancer or smooth muscle cells [19,29,41]. Additionally, miR-143 is one of the miRNAs that has been suggested to contribute to the development of T2D [30]. Both miR-143 and miR-145, induced via TGF-β, promote a transition of vascular smooth muscle cells towards a T2D-specific dysfunctional phenotype [42]. miR-143 is further induced by obesity and inhibits insulin-stimulated AKT phosphorylation in liver and adipose tissue of obese mice [30,31,32]. Both miR-143 and miR-145 have been recently reported to further interfere with insulin signaling by reducing IRS1 levels in mouse aortic smooth muscle cells [33]. We identified the miR-143/145 cluster as TGF-β-regulated in human skeletal muscle cells and found that it was significantly more induced in low responders by training. Direct overexpression in fully differentiated, multinucleated human myotubes showed that miR-143-suppressed *IRS1* and novel confirmed targets *MYH1/4*. Thus, our data suggest that part of the negative effect of TGF-ß on muscle function and insulin signaling capability is mediated by the TGF-β-induced miR-143/145 cluster.

We chose two distinct approaches for the analysis of sncRNA sequencing data. Compared to the classical approach, the main advantage of the DEUS analysis is the direct use of unique sequences [35]. In the case of our dataset, about 60% of the unique sequences were annotated to known sncRNAs. The use of the second, classical pipeline and subsequent analysis with a combination of both results validated our results. Two qPCR-confirmed regulated miRNAs, miR-21 and miR-145, were only found by DEUS analysis. The most upregulated miRNAs we found after TGF-β1 treatment, namely miR-143, miR-145, miR-181a2, and miR-31, were described as regulated by TGF-β in other tissues before [29,41,43,44]. The miRNAs we found downregulated by TGF-β1, i.e., miR-139, miR-146b, miR-208b, and miR-499a, were described in skeletal muscle differentiation or development [45,46,47,48,49]. The origin of two intragenic miRNAs lies in MYH genes: miR-208b is located within *MYH7* and miR-499a in *MYH7B*. It is not uncommon to find intragenic miRNAs, expressed with their host gene [50]. Even though miRNAs can support or antagonize expression of their host gene [51], we decided to exclude the miRNAs that were regulated alongside myotube differentiation in the study, as a specific regulation cannot be distinguished from TGF-β’s negative impact on differentiation.

Additionally, there are miRNAs that are co-expressed with other miRNAs. In our study, we found the cluster covering miR-143 and miR-145 to be of interest. Their locations in the genome are only separated by approximately 1500 bp and they are co-transcribed from the long non-coding microRNA-143 host gene [52]. This further confirms the higher sensitivity utilizing DEUS where both miR-143 and miR-145 were identified over classical approaches where only miR-143 was found. Synergistic effects of miR-143 and miR-145 were described in bladder cancer-repressing PI3K/AKT and MAPK pathways [53]. On the other hand, in the liver of mice, miR-143 inhibited insulin-stimulated AKT activation but miR-145 did not [31]. Clear suppressing effects of overexpression of miR-143 on insulin-induced phosphorylation of AKT were demonstrated in liver cells of mice [31] and vascular smooth muscle cells [32], and a mechanism via OSBPL8, also referred to as ORP8, was described in [31,32]. Thus, we were surprised to find no effects of TGF-β1 or miR-143 on OSBPL8. In contrast to Jordan et al. [31], who found nearly no expression of OSBPL8 in skeletal muscle of mice, OSBPL8 was clearly expressed in primary human myotubes in our experiments, and OSBPL8 protein levels were very stable. We, however, also found a significant reduction in insulin-induced phosphorylation of AKT by TGF-β1 treatment along with a strong induction of miR-143/145. This suggested that, in human skeletal muscle cells, suppression of insulin signaling could occur via TGF-β-mediated induction of miR-143/145 but independently of OSBPL8.

One potential target of the miR-143/145 cluster we identified was IRS1, which was previously reported to be downregulated by these miRNAs in mouse aortic smooth muscle cells [33]. The insulin receptor substrate IRS1 is a key target of the insulin receptor tyrosine kinase and is required for insulin-associated control of metabolism [54,55]. We found that IRS1 was strongly and significantly downregulated in fully differentiated human myotubes on mRNA and protein level after TGF-β1 treatment. Further, we suggest that this regulation is finetuned by an upregulation of miR-143 as the transfection of miR-143 also significantly reduced *IRS1* expression in these skeletal muscle cells. So, we, for the first time, provide evidence that directly connects TGF-β and the miR-143/145 cluster to dysregulation of insulin signaling via a downregulation of *IRS1* in human skeletal muscle cells.

TGF-β is implicated in the differentiation of smooth muscle cells (SMCs) [56,57] and this process also involves miR-143 and miR-145 [41,58,59]. However, dysregulated continuous exposure of SMCs to TGF-β appears harmful for proper differentiation, reducing the plasticity inherent to SMCs, generating a type 2 diabetic SMC phenotype. Its key features, a rhomboid morphology, F-actin fragmentation and reduced proliferation capacity, which can conceivably contribute to impaired vessel remodeling, were described to be mediated by TGF-β induced miR-143/145 [42]. Thus, dysregulated TGF-β activation in skeletal muscle of low responders might not only affect insulin signaling in myofibers, but also a proper adaptation of vessels to training which can influence the supply of substrate for skeletal muscle metabolism.

The inhibition of myo-differentiation by TGF-β was first described in 1986 [17,60]. More recently, Winbanks et al. showed that TGF-β inhibits myo-differentiation via miR-206 and miR-29 which both also downregulate HDAC4 independent of TGF-β [22]. The HDAC4 transcript was reduced after TGF-β1-treatment of myoblasts and differentiated myotubes. This is a potential target of miR-143/145; however, this could not be confirmed by miRNA mimic transfection. Other differentiation-relevant genes that appeared in our list of potential targets, due to a strong reduction by TGF-β1 in differentiating myotubes and the existence of seed sequences for miR-143/145, according to miRWalk3.0, were the myosin heavy chains. Both expression of *MYH1* and *MYH4* was significantly reduced in skeletal muscle cells after transfection of miR-143 and miR-145. Even after 96 h, the effect of miR-143 to reduce *MYH1* expression prevailed in fully differentiated skeletal muscle cells. To our knowledge, this is the first time that the potential miR-143 targets *MYH1* and *MYH4* were validated by experimental evidence. This direct regulation of MYHs by miR-143/145 appears to be one arm of TGF-β’s negative influence on skeletal muscle cell differentiation and muscular function.

We transfected primary human myotubes with miRNA mimics, which possess the sequence of the respective mature miRNA, instead of using viral transduction of vectors. To circumvent overload with RNA species, we used low miRNA concentrations; however, we still detected vast increases of miRNA levels. The use of miRNA mimics has been criticized to lead to unspecific effects, where miRNA mimics with a random sequence also caused effects [61]. Despite other shortcomings associated with viral infection systems, the use of lentiviral transduction of pre-miRNA containing plasmids was shown to affect targets at much lower measurable concentrations of mature miRNA [61]. The main difference between the two systems is the maturity state of the delivered miRNA. In lentiviral transductions, pre-miRNAs, in the form of stem loop miRNAs, are used, while miRNA mimics are based on the mature miRNA sequence. The processing of stem loop miRNAs to functional mature miRNAs involves several steps and enzymes [62]. It is, thus, arguable that the processing of miRNAs supports their functionality and transfection of miRNA mimics, bypassing parts of the miRNA machinery. Furthermore, passenger strands of miRNA mimics can accumulate in the cells and potentially lead to unspecific effects [61,63]. Additionally, big parts of miRNA mimics might not be active in the cytoplasm but packed into cellular vesicles and thereby inactivated [64]. Taking all these potential impediments into consideration further underlines and validates the reported effects for miR-143 in skeletal muscle cells by directly reducing expression of novel confirmed target *MYH1/4* and *IRS1*. The effects of miRNAs in general should be regarded as finetuning rather than major players of regulation. In a physiological system, the impact of one specific miRNA depends on both abundance of miRNA and of targets within the cell at a given timepoint, and regulatory feedback loops within the system. These time-dependent variations can be observed in our data as well when comparing target regulation after 48 h and 96 h of transfection of miRNAs. This high complexity of potential regulatory steps likely explains why we did not observe a direct impact of elevation of one or two miRNAs on insulin-dependent phosphorylation of AKT but could merely report on the regulation of insulin signaling molecule expression by these miRNAs. To achieve meaningful effects within a physiological system, a whole class of specific miRNAs, including the here identified miR-143, needs to be taken into consideration. Finding these additional TGF-β-regulated factors that are able to modulate glycemic control in human skeletal muscle would be of interest in future investigations.

Finally, we speculated that the impaired response in insulin sensitivity after a training intervention is the result of an interplay between organs, caused by circulating miRNAs which possibly originate from dysregulated TGF-β signaling in skeletal muscle after exercise. Possibly, circulating miRNAs influence other tissues, for example liver or adipose tissue. Interestingly, many of the miRNAs regulated by TGF-β were described to be affected by exercise, including differentiation-independent and differentiation-dependent miRNAs. Described exercise-associated circulating microRNAs that were regulated by TGF-β in our experiments are miR-21, miR-133b, miR-143, miR-145, miR-146, miR-206, and miR-499 [65]. It has been shown that miR-143 impairs insulin signaling in liver [31]. To test a possible skeletal muscle-liver cross-talk, we measured miR-143/145 levels in blood samples of human individuals before and after 8 weeks of exercise intervention. While TGF-β signaling and miR-143/145 were significantly more induced by training in the skeletal muscle of individuals who did not improve insulin sensitivity, a systemic regulation of these miRNAs was not detected. Thus, if messenger miRNAs transport the information of TGF-β dysregulation from the exercising muscle to other organs, they have yet to be identified.

## 5. Conclusions

Our results clearly associate TGF-β signaling and its regulation of the miR-143/145 cluster in skeletal muscle with a lack of a beneficial outcome regarding improved glycemic control after training. We identified the TGFβ/miR-143/145 axis in skeletal muscle cells and showed that the negative effect of elevated TGF-β signaling, namely reducing insulin signaling and muscular function, is finetuned via TGF-β-dependent upregulation of miR-143. Elevated TGF-β signaling and miR-143/145 induction in LREs might obstruct improvements in insulin sensitivity by training in two ways. On the one hand, by directly impairing insulin signaling via a reduction in INSR by TGF-β and a finetuned suppression of *IRS1* by miR-143. On the other hand, the observed suppression of novel targets *MYH1* and *MYH4* by TGF-β-induced miR-143 might negatively impact muscle cell fusion and muscular functionality. Identification of the precise mechanisms, by which activation of TGF-β signaling, observed in low responders, impacts insulin sensitivity, provides ground for the development of novel strategies to improve glycemic control in patients with risk for T2D.

## Figures and Tables

**Table 1:** Participant characteristics.

**Sex**	25 females/15 males	
**Age**	36.84 ± 12.23	Years
**WHR**	0.90 ± 0.05	cm/cm
**BMI**	31.68 ± 4.39	kg/m^2^
**Fasting glucose**	5.29 ± 0.45	mmol/L
**Fasting insulin**	93.52 ± 40.25	pmol/L
**VO_2_peak**	25.42 ± 4.82	ml/min∗kg
**HbA1c**	35.03 ± 3.40	mmol/mol Hb

WHR = waist to hip ratio.

**Table 2:** Regulation of potential miR-143/145 cluster targets by TGF-β1 in differentiated skeletal muscle cells.

		TGF-ß	*p* vs. Ctrl	TGF-ß + SB	*p* vs. TGF-ß
**Differentiation**	***MYH1***	0.12	1.32 × 10^−5^	0.99	1.86 × 10^−5^
	***MYH2***	0.03	4.73 × 10^−6^	1.22	4.11 × 10^−7^
	***MYH4***	0.04	6.01 × 10^−8^	0.96	1.40 × 10^−7^
	***MYH7***	0.03	8.40 × 10^−8^	0.78	5.15 × 10^−6^
**Targets**	***ARPP21***	0.35	1.80 × 10^−9^	0.72	3.18 × 10^−6^
	***HDAC4***	0.41	8.07 × 10^−6^	0.84	5.44 × 10^−4^
	***HDAC9***	0.21	3.03 × 10^−6^	1.12	1.81 × 10^−6^
	***INSR***	0.44	5.88 × 10^−8^	0.76	5.90 × 10^−5^
	***IRS1***	0.42	1.06 × 10^−7^	0.98	2.21 × 10^−7^
	***OSBPL8***	1.46	4.22 × 10^−5^	0.94	8.71 × 10^−6^
	***PLEKHB2***	0.87	n.s.	1.02	n.s.
	***TP63***	0.15	6.69 × 10^−6^	0.59	7.00 × 10^−3^

Primary human myoblasts (*n* = 6) were differentiated towards myotubes for 5 days in the absence of FBS and subsequently treated with 1 ng/mL of TGF-β1 (TGFβ), TGF-β1 plus inhibitor (10 µM of SB431542) (TβSB), or vehicle control (Ctrl) for 48 h. Expression of mature miRNA miR-143-3p, miR-145-5p, and mRNA levels of potential targets were detected by qPCR. All qPCR data were normalized to individual donors and reference RNU6 (miRNAs) or mean of *RPS28* and *TBP* (mRNA). The mean fold change compared to Ctrl is listed. Statistical analyses were performed on rel. expression values using one-way ANOVA with Fisher’s LSD post-hoc test, *p* < 0.05.

**Table 3:** Regulation of potential targets by miR-143/145 cluster in differentiated skeletal muscle cells.

		miR143	*p* vs. Ctrl	miR145	*p* vs. Ctrl	miR143 + 145	*p* vs. Ctrl
**Differentiation**	***MYH1***	0.79	0.0326	0.77	0.0325	0.79	0.0293
	***MYH2***	0.88	n.s.	0.90	n.s.	0.96	n.s.
	***MYH4***	0.77	0.0021	0.70	0.0002	0.78	0.0033
	***MYH7***	0.93	n.s.	1.02	n.s.	0.91	n.s.
**Targets**	***ARPP21***	0.86	n.s.	0.88	n.s.	0.84	0.0479
	***HDAC4***	0.87	n.s.	1.11	n.s.	0.96	n.s.
	***HDAC9***	0.93	n.s.	0.94	n.s.	0.98	n.s.
	***INSR***	0.87	n.s.	1.00	n.s.	0.97	n.s.
	***IRS1***	0.74	0.0302	0.99	n.s.	0.70	0.0287
	***OSBPL8***	1.02	n.s.	1.01	n.s.	1.06	n.s.
	***PLEKHB2***	1.02	n.s.	1.04	n.s.	1.00	n.s.
	***TP63***	0.76	0.0121	0.91	n.s.	0.91	n.s.

Primary human myoblasts (*n* = 5) were differentiated towards myotubes for 5 days in the absence of FBS and subsequently transfected with miR-143-3p, miR-145-5p, a combination of miR-143-3p and miR-145-5p, control miR-122-5p, or vehicle control (Ctrl) for 48 h. Expression of mature miRNA miR-143-3p, miR-145-5p, and mRNA levels of potential targets were detected by qPCR. All qPCR data were normalized to individual donors and reference RNU6 (miRNAs) or mean of *RPS28* and *TBP* (mRNA). The mean fold change compared to Ctrl is listed. Statistical analyses were performed on rel. expression values using one-way ANOVA with Fisher’s LSD post-hoc test, *p* < 0.05.

## Data Availability

The data presented in this study are available on request from the corresponding author.

## Supplementary Materials

The following are available online at https://www.mdpi.com/article/10.3390/cells10123443/s1, Figure S1. Improved myotube differentiation at complete absence of FBS, Figure S2. TGF-β regulates miR-143-3p/145-5p and miR-181a2-5p in myoblasts, Figure S3. No miR-181a2-5p induction in low-responders with elevated TGF-β signaling by training, Figure S4. Correlation of miR-143/145 and potential skeletal muscle specific target fold changes after training, Figure S5. miR-143/145 downregulate components of skeletal muscle differentiation after 96 h, Table S1. Regulation of potential targets by miR-143/145 in differentiated skeletal muscle cells after 96 h, Table S2. Primer Assays and Antibodies.
