# Supplementary material for: TGF-β Induction of miR-143/145 Is Associated to Exercise Response by Influencing Differentiation and Insulin Signaling Molecules in Human Skeletal Muscle
Source: Cells. 2021 Dec 7;10(12):3443. doi: 10.3390/cells10123443 (PMC8700369; doi:10.3390/cells10123443)
Supplement: Supplementary file 1 [file cells-10-03443-s001.zip › cells-1473823-supplementary.pdf]

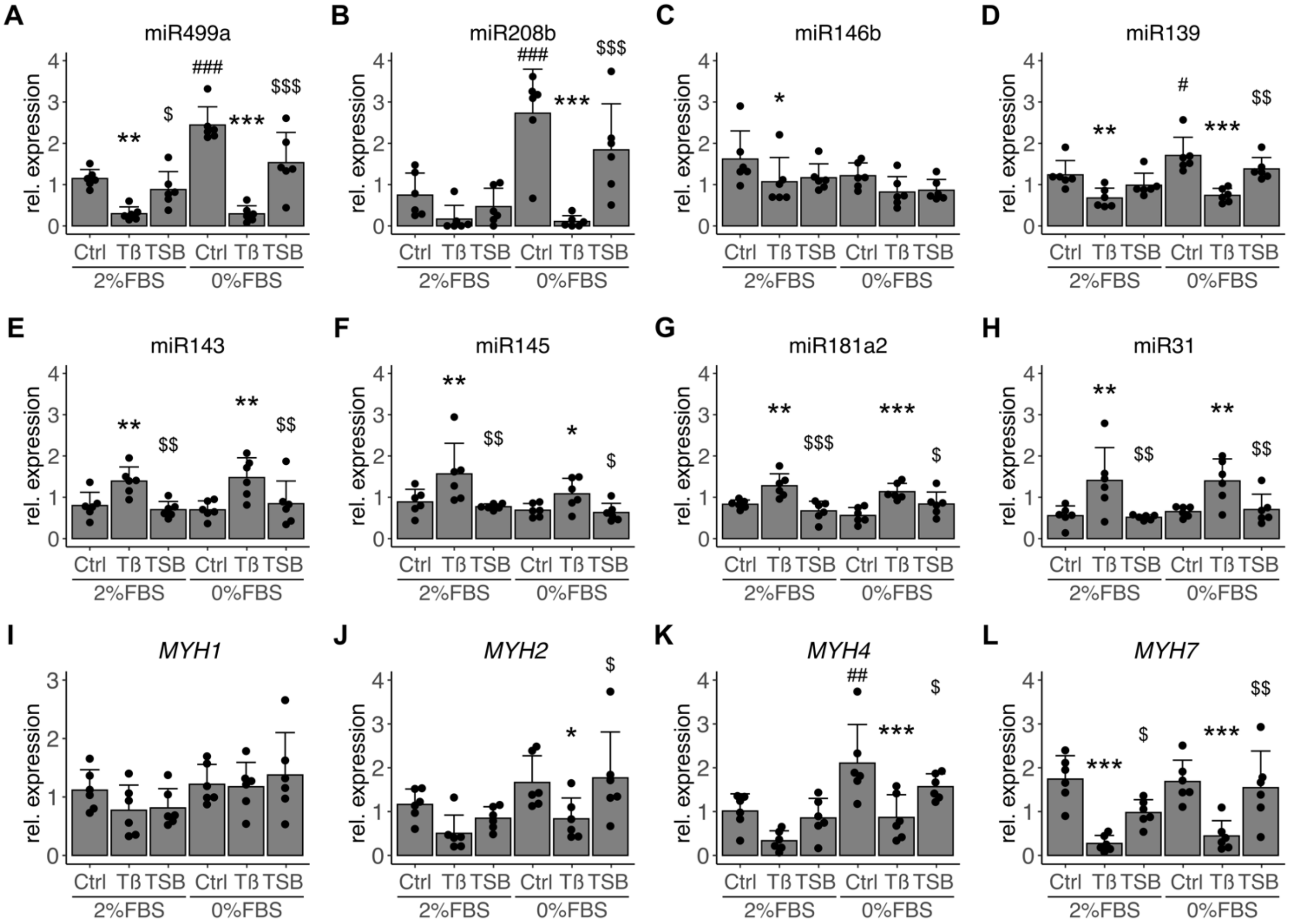

Figure S1. Increased expression of muscle-specific RNAs in myotubes differentiated at complete absence of FBS Primary human myoblasts (n=6) were treated with 1 ng/ml TGF-β1 (Tβ), TGF-β1 plus inhibitor (10 μM SB431542) (TSB), or vehicle control (Ctrl) for 48 h. Subsequently, myoblasts were differentiated towards myotubes for 5 days in the presence (2%) or absence (0%) of FBS. Expression of mature miRNA (A) miR-499a-5p, (B) miR-208b, (C) miR-146b-5p, (D) miR-139-5p, (E) miR-143-3p, (F) miR-145-5p, (G) miR-181a2-5p, (H) miR-31-5p and mRNA (I) *MYH1*, (J) *MYH2*, (K) *MYH4*, (L) *MYH7* was detected by qPCR. All qPCR data were normalized to individual donors and reference RNU6 (miRNAs) or mean of *RPS28* and *TBP* (mRNA). Individual datapoints are displayed, bars represent mean ± SD. All data were analyzed using one-way ANOVA with Bonferroni correction, * $p<0.05$, ** $p<0.01$, *** $p<0.001$ vs Ctrl at same FBS concentration, $ $p<0.05$, $$ $p<0.01$, $$$ $p<0.001$ vs Tβ at same FBS concentration, # $p<0.05$, ## $p<0.01$, ### $p<0.001$ 0% vs 2% FBS.

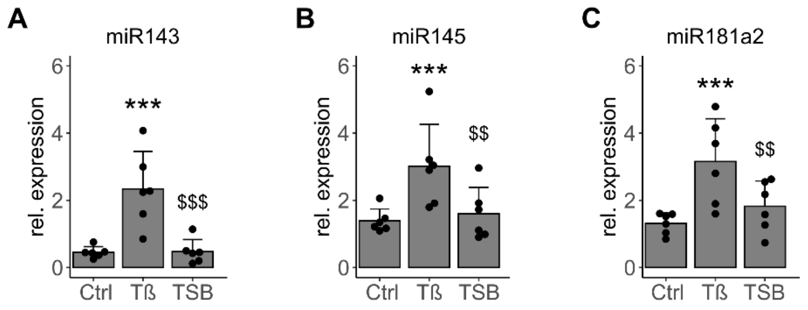

Figure S2. TGF-β regulates miR-143-3p/145-5p and miR-181a2-5p in myoblasts
Primary human myoblasts (n=6) were treated with 1 ng/ml TGF-β1 (Tβ), TGF-β1 plus inhibitor (10 μM SB431542) (TSB), or vehicle control (Ctrl) for 48 h. Expression of mature miRNA (A) miR-143-3p, (B) miR-145-5p, (C) miR-181a2-5p was detected by qPCR. All qPCR data were normalized to individual donors and reference RNU6. Individual datapoints are displayed, bars represent mean ± SD. All data were analyzed using one-way ANOVA with Bonferroni correction, *** p<0.001 vs Ctrl, $$ p<0.01, $$$ p<0.001 vs Tβ.

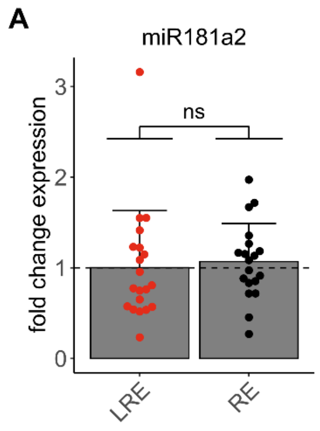

Figure S3. No miR-181a2-5p induction in low-responders with elevated TGF-β signaling by training
Middle aged sedentary individuals (n=40) were grouped into low-responders (LRE, red) and responders (re, black) based on their improvement (fold change > 1.1) in insulin sensitivity (ISIMats) after 8 weeks of training intervention. (A) Expression of mature miRNA miR-181a2-5p was detected in muscle biopsies by qPCR, normalized to individual donors and reference RNU6. Individual datapoints are displayed, bars represent mean ± SD. Data were analyzed using one-way ANOVA with Fisher´s LSD post-hoc test.

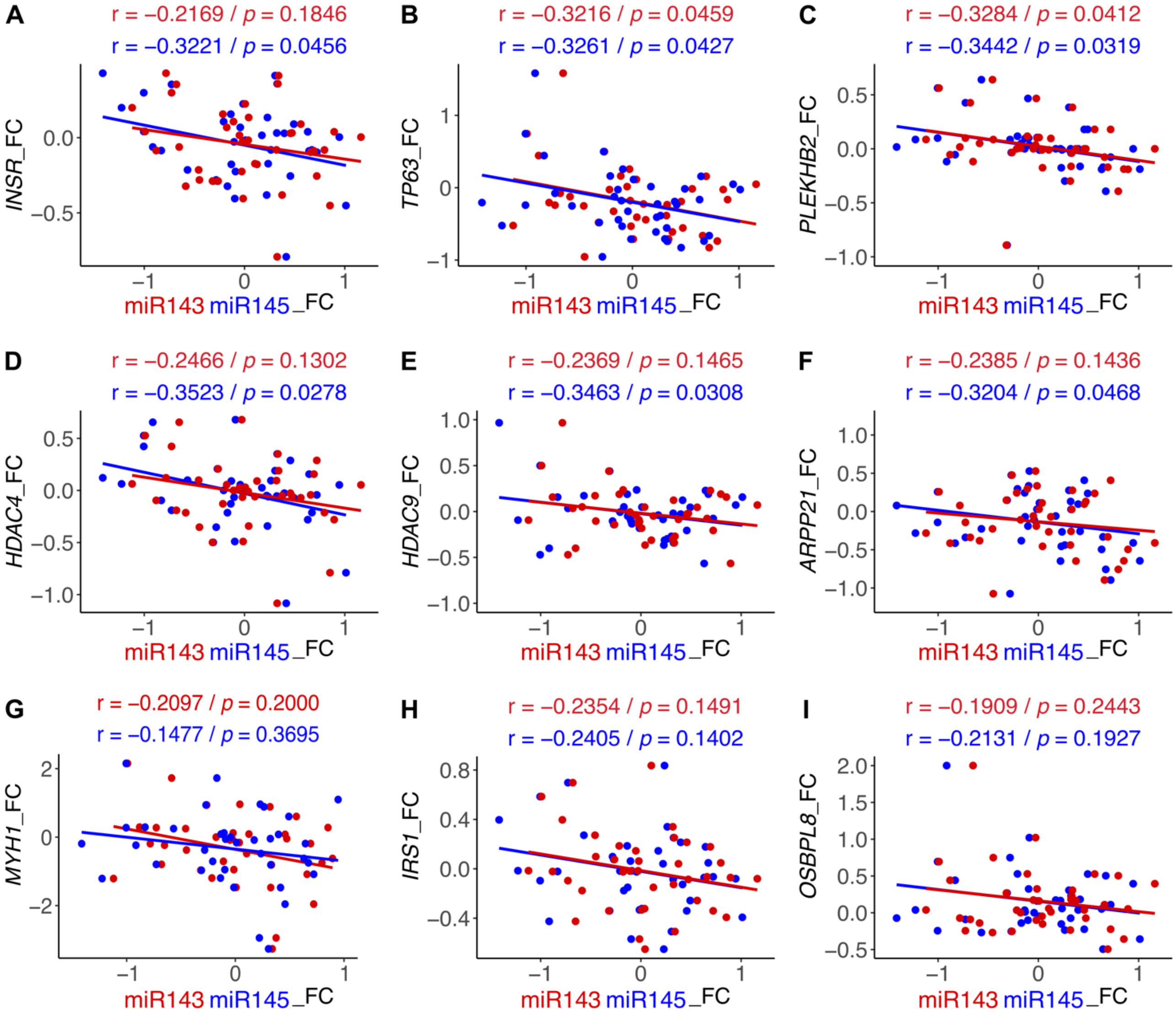

Figure S4. Correlation of miR-143/145 and potential skeletal muscle specific target fold changes after training Muscle biopsies were taken from middle aged sedentary individuals (n=40) before and after 8 weeks of training intervention. RNA isolated from muscle biopsies was used for transcriptome analyses and detection of miRNAs via qPCR. (A-I) qPCR data were normalized to individual donors and reference RNU6. Correlation analyses were performed between fold changes (after intervention vs. before intervention) of miR-143-3p/145-5p and potential targets. Data were analyzed using Pearson correlation, coefficient r and p-values are indicated.

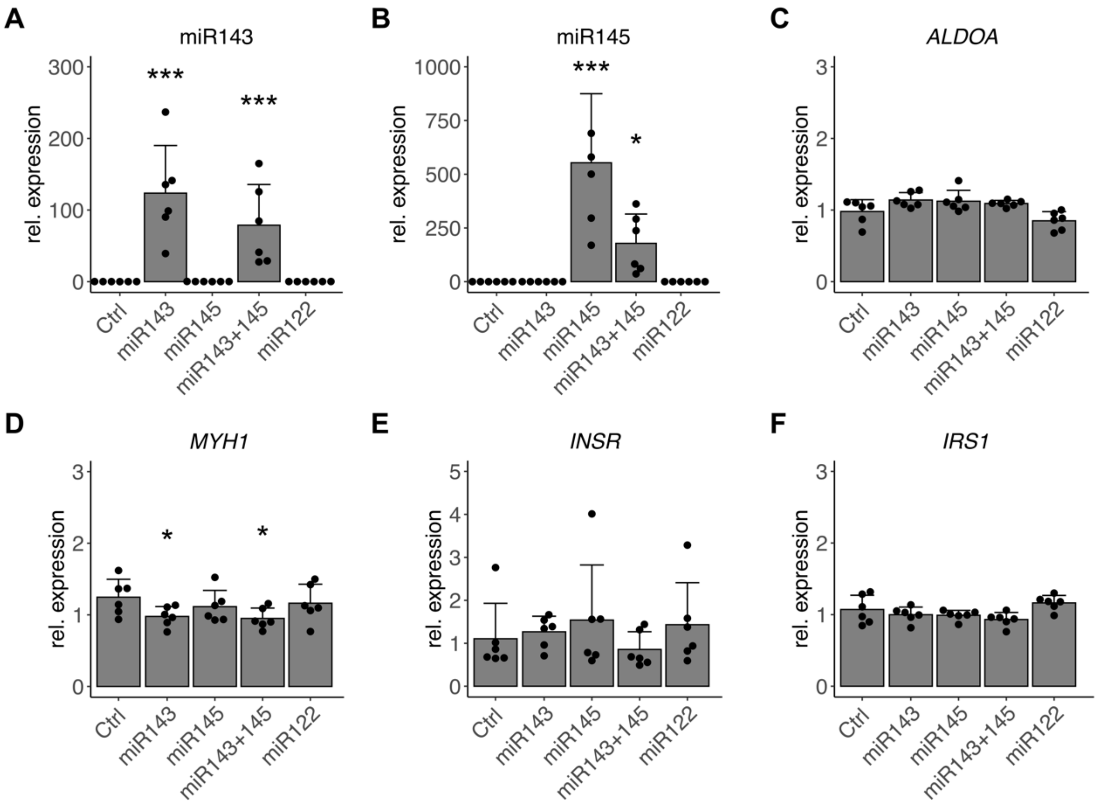

Figure S5. miR-143/145 downregulate components of skeletal muscle differentiation after 96 h
Primary human myoblasts (n=6) were differentiated towards myotubes for 5 days in the absence of FBS and subsequently transfected with miR-143-3p, miR-145-5p, a combination of miR-143-3p and miR-145-5p, control miR-122-5p, or vehicle control (Ctrl) for 96 h. Expression of mature miRNA (A) miR-143-3p, (B) miR-145-5p was detected by qPCR. (C) mRNA levels of (C) miR-122-5p target *ALDOA*, (D) *MYH1*, (E) *INSR* and (F) *IRS1* were detected by qPCR. All qPCR data were normalized to individual donors and reference RNU6 (miRNAs) or *RPS28* and *TBP* (mRNA). Individual datapoints are displayed, bars represent mean ± SD. All data were analyzed using one-way ANOVA with Fisher´s LSD post-hoc test, * $p<0.05$, *** $p<0.001$ vs Ctrl.

		miR143	p vs. Ctrl	miR145	p vs. Ctrl	miR143+145	p vs. Ctrl
**Differentiation**	***MYH1***	0.82	0.0304	0.91	n.s.	0.79	0.0179
	MYH2	1.07	n.s.	0.96	n.s.	0.92	n.s.
	MYH4	0.98	n.s.	0.93	n.s.	0.95	n.s.
	MYH7	0.94	n.s.	0.95	n.s.	1.07	n.s.
**Targets**	***ARPP21***	0.96	n.s.	0.84	0.0088	0.98	n.s.
	FNBP1	1.19	n.s.	1.29	0.0254	1.12	n.s.
	HDAC4	1.18	n.s.	1.14	n.s.	1.16	n.s.
	HDAC9	1.38	n.s.	1.08	n.s.	1.40	n.s.
	INSR	1.50	n.s.	1.88	n.s.	1.11	n.s.
	IRS1	0.97	n.s.	0.95	n.s.	0.90	n.s.
	OSBPL8	1.07	n.s.	1.03	n.s.	1.09	n.s.
	PLEKHB2	1.05	n.s.	0.97	n.s.	1.01	n.s.
	TP63	0.84	0.0481	1.01	n.s.	0.89	n.s.

Table S1. Regulation of potential targets by miR-143/145 in differentiated skeletal muscle cells after 96 h Primary human myoblasts (n=6) were differentiated towards myotubes for 5 days in the absence of FBS and subsequently transfected with miR-143-3p, miR-145-5p, a combination of miR-143-3p and miR-145-5p, control miR-122-5p, or vehicle control (Ctrl) for 96 h. Expression of mature miRNA miR-143-3p, miR-145-5p and mRNA levels of potential targets were detected by qPCR. All qPCR data were normalized to individual donors and reference RNU6 (miRNAs) or mean of RPS28 and TBP (mRNA). The mean fold change compared to Ctrl is listed. Statistical analyses were performed on rel. expression values using one-way ANOVA with Fisher´s LSD post-hoc test, $p<0.05$.

RNA	Primer	Cat. Nr.
ARPP21	Hs_ARPP21_3_SG	QT01192562
FNBP1	Hs_FNBP1_1_SG	QT00030198
HDAC4	Hs_HDAC4_1_SG	QT00005810
HDAC9	Hs_HDAC9_1_SG	QT00039333
INSR	Hs_INSR_1_SG	QT00082810
IRS1	Hs_IRS1_1_SG	QT00074144
MYH1	Hs_MYH1_2_SG	QT01671005
MYH2	Hs_MYH2_1_SG	QT00082495
MYH4	Hs_MYH4_2_SG	QT01668779
MYH7	Hs_MYH7_1_SG	QT00000602
OSBPL8	Hs_OSBPL8_1_SG	QT00067102
PLEKHB2	Hs_PLEKHB2_1_SG	QT00082936
TP63	Hs_TP63_2_SG	QT02424051
RPS28	Hs_RPS28_2_SG	QT02310203
TBP	Hs_TBP_1_SG	QT00000721
**miR-31**	hsa-miR-31-5p	MS00003290 / 002279
**miR-139**	hsa-miR-139-5p	MS00003493
**miR-143**	hsa-miR-143-3p	MS00003514 / 002249
**miR-145**	hsa-miR-145-5p	MS00003528 / 002278
**miR-146b**	hsa-miR-146b-5p	MS00003542
**miR-181a2**	hsa-miR-181a-2-3p	MS00008834 / 000480
**miR-208b**	hsa-miR-208b	MS00009058
**miR-499a**	hsa-miR-499a-5p	MS00004375
**RNU6**	RNU6	MS00033740

Protein	Antibody	Cat. Nr.
**pAKT[S473]**	Cell Signaling	9271L
**tAKT**	BD	610860
**INSR**	Cell Signaling	3025
**IRS1**	Millipore	06-248
**MyHfast**	Sigma Clone MY-32	M4276
**OSBPL8**	Abcam	AB60110
**GAPDH**	Abcam	AB8245
**donkey anti-rabbit**	Licor	926-68023
**donkey anti-mouse**	Licor	p26-68022
**goat anti-mouse**	Licor	925-68070
**goat anti-rabbit**	Licor	926-32211

Table S2. Primer Assays and Antibodies
Indicated miRNAs were measured with either Qiagen/TaqMan Assays.
